# Inhibition of STAT3 by S3I‐201 suppress peritoneal fibroblast phenotype conversion and alleviate peritoneal fibrosis

**DOI:** 10.1111/jcmm.18381

**Published:** 2024-05-23

**Authors:** Qianhui Song, Han Li, Hao Yan, Zanzhe Yu, Zhenyuan Li, Jiangzi Yuan, Na Jiang, Zhaohui Ni, Leyi Gu, Wei Fang

**Affiliations:** ^1^ Department of Nephrology, Renji Hospital, School of Medicine Shanghai Jiao Tong University Shanghai People's Republic of China; ^2^ Shanghai Center for Peritoneal Dialysis Research Shanghai People's Republic of China

**Keywords:** fibroblasts, fibrosis, peritoneal dialysis, S3I‐201, signal transducer and activator of transcription 3

## Abstract

Peritoneal fibrosis is a common pathological response to long‐term peritoneal dialysis (PD) and a major cause for PD discontinuation. Understanding the cellular and molecular mechanisms underlying the induction and progression of peritoneal fibrosis is of great interest. In our study, in vitro study revealed that signal transducer and activator of transcription 3 (STAT3) is a key factor in fibroblast activation and extracellular matrix (ECM) synthesis. Furthermore, STAT3 induced by IL‐6 trans‐signalling pathway mediate the fibroblasts of the peritoneal stroma contributed to peritoneal fibrosis. Inhibition of STAT3 exerts an antifibrotic effect by attenuating fibroblast activation and ECM production with an in vitro co‐culture model. Moreover, STAT3 plays an important role in the peritoneal fibrosis in an animal model of peritoneal fibrosis developed in mice. Blocking STAT3 can reduce the peritoneal morphological changes induced by chlorhexidine gluconate. In conclusion, our findings suggested STAT3 signalling played an important role in peritoneal fibrosis. Therefore, blocking STAT3 might become a potential treatment strategy in peritoneal fibrosis.

## 1 INTRODUCTION

Peritoneal dialysis (PD) is a well‐established home dialysis therapy for patients with end‐stage kidney disease (ESKD). However, continuous exposure to bio‐incompatible PD solutions, uremic toxins, peritonitis, etc. lead to the alterations of peritoneal membrane structure and function.^1^ The most common morphological alterations of peritoneum after long‐term PD include loss of the mesothelial cell layer, neoangiogenesis and thickening of the submesothelial area with fibroblasts activation and augmentation of collagen fibres.^1^, ^2^ Peritoneal fibrosis is the final common pathway of progressive peritoneal injury leading to membrane dysfunction and remains a serious complication occurred after prolonged PD, but clinically, effective therapies for peritoneal fibrosis are currently not available. Therefore, to develop new strategies for anti‐peritoneal fibrosis therapy is urgently required.

The mechanisms that initiate and sustain peritoneal fibrosis during PD are still under fully understood. Myofibroblasts are the main effector cells that synthesize and secrete extracellular matrix (ECM) components during fibrosis, and the activation of fibroblasts to be transformed into α‐smooth muscle actin (α‐SMA) positive myofibroblasts is the critical step to initiate fibrosis. In recent years, the paradigm that the mesothelial‐mesenchymal transition (MMT) of mesothelial cells (MCs) was the first step in the evolution of peritoneal fibrosis has been reconsidered.^3^, ^4^ Similar to renal fibrosis, lineage tracing studies have shown that myofibroblasts found in the fibrotic peritoneum have different sources, including submesothelial fibroblasts, MCs, pericytes, bone marrow derived cells and adipocytes etc., whereas submesothelial fibroblasts rather than mesothelial cells are the conceivable main source of collagen producing myofibroblasts that drives fibrogenesis.^4^, ^5^, ^6^ However, there are few studies have investigated the functional evolution of fibroblasts during the process of peritoneal fibrosis.

Intraperitoneal inflammation has been demonstrated to be the main cause of the initiation and maintenance of peritoneal fibrosis.^7^ We previously reported that IL‐6 trans‐signalling activates signal transducer and activator of transcription 3 (STAT3) in human peritoneal mesothelial cells (HPMCs) and participate in the MMT process to promote peritoneal fibrosis.^8^ STAT3 is an important transcription factor and a transducer of multicellular surface receptors that regulates many downstream target genes.^9^ Increasing evidence has suggested that STAT3 was a critical regulator of fibroblasts phenotype conversion and deposition of ECM.^10^, ^11^ In cardiac fibroblasts, activation of STAT3 has been shown to be associated with a phenotypic conversion from fibroblasts to myofibroblasts as well as the synthesis of ECM components.^12^ Recently, Chakraborty et al. reported that STAT3‐deficient fibroblasts are less sensitive to the pro‐fibrotic effects of TGF‐β and fibroblast‐specific knockout of STAT3, or its pharmacological inhibition, ameliorate skin fibrosis in experimental mouse models.^13^ S3I‐201 (NSC 74859), a chemical probe inhibitor of STAT3 activity, which inhibits STAT3‐STAT3 complex formation, STAT3‐DNA binding and transcriptional activities,^14^ has been reported to suppress fibrogenesis in renal,^11^ liver^15^ and pulmonary fibrosis.^16^ However, current studies on the role of STAT3 during fibroblasts in peritoneal fibrosis remains unclear.

In this study, we investigated the role of STAT3 in fibroblasts phenotype conversion and the effect of pharmacological blockade of STAT3 in the development of peritoneal fibrosis.

## 2 MATERIALS AND METHODS

### 2.1 Ethics statement

The study protocol conformed with the ethical guidelines of the 1975 Declaration of Helsinki and was approved by the Ethics Committee of Renji Hospital. The experimental protocols strictly complied with the institutional guidelines and the criteria outlined in the National Institutes of Health Guide for the Care and Use of Laboratory Animals (NIH Pub. No. 80‐23) and was approved by Renji Hospital, School of Medicine, Shanghai Jiao Tong University.

### 2.2 Reagents and antibodies

Antibodies to p‐STAT3 (#9145), STAT3 (#4904), GAPDH (#5174) were purchased from Cell Signaling Technology (Danvers, USA). Antibodies to Cytokeratin (#10830‐1‐AP), Vimentin (#10366), Collagen I (#14695), α‐SMA (#14395) and CD45 (#20103‐1‐AP) were purchased from Proteintech (Rosemont, USA). Anti‐Factor VIII (#NB100‐91761) was from Novus Biologicals (Centennial, USA). Dispase II was purchased from Roche (Mannheim, Germany). Recombinant rat IL‐6 protein (#506‐RL), mouse IL‐6R protein (#1830‐SR), rat gp130 Fc protein (#5029‐RG) and mouse TGF‐beta 1 protein (#7666‐MB) were purchased from R&D Systems (Minneapolis, USA). The STAT3‐specific inhibitor S3I‐201 (#SML0330) and CG (#C9394) were purchased from Sigma‐Aldrich (Darmstadt, Germany). For in vivo studies, S3I‐201 was further diluted with the medium and the concentration of DMSO was below 0.001%. For in vitro studies, S3I‐201 was further diluted with PBS and the concentration of DMSO was below 0.01%. DMEM/F12 and other cell culture reagents were obtained from Gibco Corp (Carlsbad, USA).

### 2.3 RPMC isolation and cell culture

Primary rat peritoneal mesothelial cells (RPMCs) were isolated from rat peritoneum with dispase digestion.^17^, ^18^ Briefly, a Sprague–Dawley rat was anaesthetised and its fur on the abdomen was shaved off to expose the abdominal cavities. The entire peritoneum was isolated under sterile conditions and planted into a 6 cm cell culture dishes, then digested with Dispase II solution (2U/ml in DMEM/F12) in an atmosphere of 5% CO2 and 95% air at 37°C for 1 h. Cells were harvested using a cell scraper to gently scrape the parietal peritoneal surface and the media containing the cells were centrifuged at 1, 000 rpm for 10 min at RT. Then cells were gently resuspended in DMEM/F12 containing 10% FBS and 0.1% penicillin–streptomycin solution. The medium was replaced 24 h later at first and then replaced every 3 days. Cells were cultured for about 7 days until they were at 80%–90% confluency. When confluent, the cells were washed twice in PBS and detached using 0.05% trypsin at 37°C for 5 min. Characteristic of cultured RPMCs was determined by immunofluorescence staining with antibodies against cytokeratin and vimentin. The purity of RPMCs reached to 90%. The third to fifth generation RPMCs were used for experiments.

### 2.4 RFB isolation and cell culture

Primary rat peritoneal fibroblasts (RPFBs) were isolated as previously reported.^19^ Briefly, a Sprague–Dawley rat was anaesthetised and its fur on the abdomen was shaved off to expose the abdominal cavities. The peritoneal cavities were then opened and the entire peritoneum was isolated under sterile conditions. The peritoneum was minced using sterile scalpel into 1 mm^3^ pieces and planted into a 25 cm^2^ cell culture flasks in an atmosphere of 5% CO2 and 95% air at 37°C. DMEM/F12 containing 10% FBS and 0.1% penicillin–streptomycin solution were added to the culture flasks 2 h later and refreshed the medium every 3 days. When cell outgrowth from the explants began, the remaining tissue was removed. When confluent, the cells were washed twice in PBS and detached using 0.05% trypsin at 37°C for 5 min. Characteristic of cultured RPFs was determined by immunofluorescence staining with antibodies against FSP‐1 and vimentin. The purity of RPFs reached to 95%. The third to fifth generation RPFs were used for experiments.

### 2.5 Coculture system of RPMCs and RFBs

RPMCs were initially cultured in DMEM/F12 medium with 10% FBS and 0.1% penicillin–streptomycin solution on the membrane of a transwell (Corning, USA) before the experiments. RPFBs were seeded into the lower chamber, and the RPMCs cultured on the membrane of the transwell were added to form the coculture system. The RPFBs in the lower chamber of the coculture system were collected for further experiments.

### 2.6 Establishment of a mouse peritoneal fibrosis model and S3I‐201 administration

Eight‐week‐old male C57/BL6 (*n* = 18) mice weighing 20–25 g were used in this study. The mice were housed at a constant temperature (23 ± 2°C) under a 12‐h light/dark cycle. They were fed a standard diet and distilled water. Mice were randomly assigned to three groups (*n* = 6): the peritoneal fibrosis group received intraperitoneally injections of 10 mL/kg 0.1% chlorhexidine gluconate (CG) in 15% ethanol dissolved in PBS every other day for 3 weeks, whereas mice that received intraperitoneally injections of the same dose of PBS every other day for 3 weeks served as sham controls. To investigate the effect of STAT3 inhibition on peritoneal fibrosis, S3I‐201 was injected intraperitoneally at a dose of 10 mg/kg per mouse daily based on the previous studies.^20^ The treatment group was administered 0.1% CG every other day for 3 weeks, together with S3I‐201 10 mg/kg every day for 3 weeks. The injection was performed in the mouse's left‐lower abdomen wall injection. All mice were euthanized after 3 weeks and the samples of parietal peritoneum except the injection points were collected for subsequent experiments.

### 2.7 Western blot analysis

Total protein was harvested by cracking cultured cells or peritoneal tissues using RIPA buffer (Santa Cruz, Dallas, USA). The total protein concentrations were measured via bicinchoninic acid (BCA) assay kit (Thermo Fisher, Carlsbad, USA). Denatured proteins (30–50 μg per sample) were, separated on SDS‐PAGE gel and then transferred to PVDF membranes (Bio‐Rad, Hercules, USA). Membranes were blocked with 5% milk dissolved in TBST for 1 h and incubated with primary antibody against GAPDH (dilution 1:4000), collagen‐I (dilution 1:1000), α‐SMA (dilution 1:2000), P‐STAT3 (dilution 1:2000), STAT3 (dilution 1:2000), β‐tubulin (dilution 1:4000) at 4°C overnight. After washing with TBST three times, membranes were incubated with HRP‐conjugated secondary antibodies (Beyotime, Shanghai, China) for 1 h at room temperature. The bands obtained were visualized by chemiluminescence detection system (Tanon, Shanghai, China) and analysed using the ImageJ 1.43 software (National Institutes of Health, USA).

### 2.8 Immunofluorescence

After being fixed with 4% paraformaldehyde for 10 min, all samples were permeabilized with 0.1% Triton X‐100 for 3 min and then blocked by 5% donkey serum in PBS at room temperature for 1 h. Then, primary antibodies against Cytokeratin (diluted 1:400), Vimentin (diluted 1:200), CD45 (diluted 1:100), Factor VIII (diluted 1:100) and FSP‐1 (diluted 1:200) were added to the samples and incubated overnight at 4°C. The secondary antibodies used were Alexa Fluor® 594 (Abcam, ab150116) or Alexa Fluor® 488 (Abcam, ab150077) diluted in PBS for 1 h at room temperature (diluted 1:200). The nuclei were stained with DAPI (Life Technology, Carlsbad, USA). After additional washing for 5 min three times, the images of slides were then obtained using a fluorescence microscope (ZEISS, Axio Vert A1).

### 2.9 Histological analysis of the peritoneum

A paraformaldehyde‐fixed peritoneum tissue was embedded in paraffin and sectioned to 5‐μm‐thick paraffin sections. According to the manufacturer protocol (Sigma‐Aldrich, Darmstadt, Germany), haematoxylin and eosin and Masson trichrome staining were performed to evaluate the severity of peritoneal fibrosis. Immunohistochemical staining was performed on the basis of the procedure described in previous studies.^8^, ^21^ Briefly, after deparaffinization and rehydration, paraffin sections were placed in antigen retrieval buffer (EDTA buffer (Dako; 0.25 mM, pH 8)) for heat treatment in water bath at 98°C for 20 min. Endogenous peroxidases were deactivated by incubating in peroxidase‐blocking solution at room temperature for 15 min. The paraffin sections were then incubated with the primary antibodies anti‐mouse Collagen I (diluted 1:200) and F4/80 (diluted 1:100) for 1 h at room temperature, followed by incubation with the secondary antibody and counterstaining haematoxylin. Images were obtained using a Zeiss 710 Duo microscope. ImageJ software (National Institute of Health, USA) was used to measure the positive area of peritoneum tissue.

### 2.10 Statistical analyses

Data were expressed as the mean ± SEM and all the experiments were independently repeated at least three times. The differences between two groups were determined by unpaired t‐tests. Multiple group comparisons were assessed by one‐way ANOVA. *p* < 0.05 was considered statistically significant. All data analyses were performed using the GraphPad Prism (version 8.0.0 GraphPad, San Diego, USA).

## 3 RESULTS

### 3.1 Identification of primary rat peritoneal mesothelial cells and fibroblast cells

**FIGURE 1:**
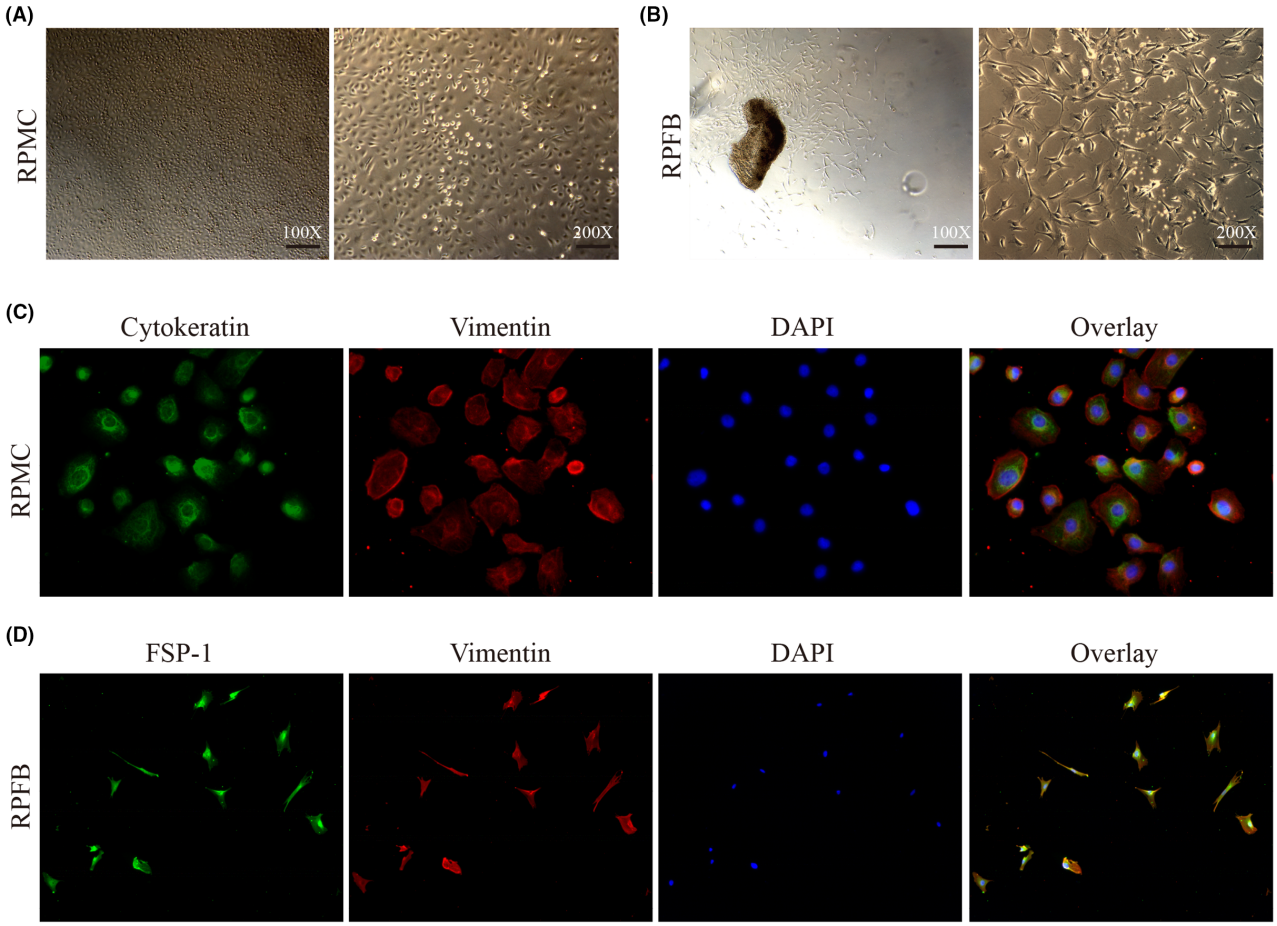
Identification of primary cultured rat peritoneal mesothelial cells and fibroblasts (100×, 200×).

As shown in Figure 1A,C, after three passages, the cells showed typically cobblestone like and were phenotypically characterized as mesothelial cells by immunofluorescence, which co‐expressed both epithelial marker cytokeratin and mesenchymal marker vimentin, negatively expressed CD45 and factor VIII (Data S1). As shown in Figure 3B,D, the cells showed typical fibroblast morphology, being positive for FSP‐1 and vimentin, and negative for α‐SMA, CD45 and factor VIII (Data S2).

### 3.2 Inhibition of STAT3 in RPFBs suppressed collagen‐I synthesis and α‐SMA expression

**FIGURE 2:**
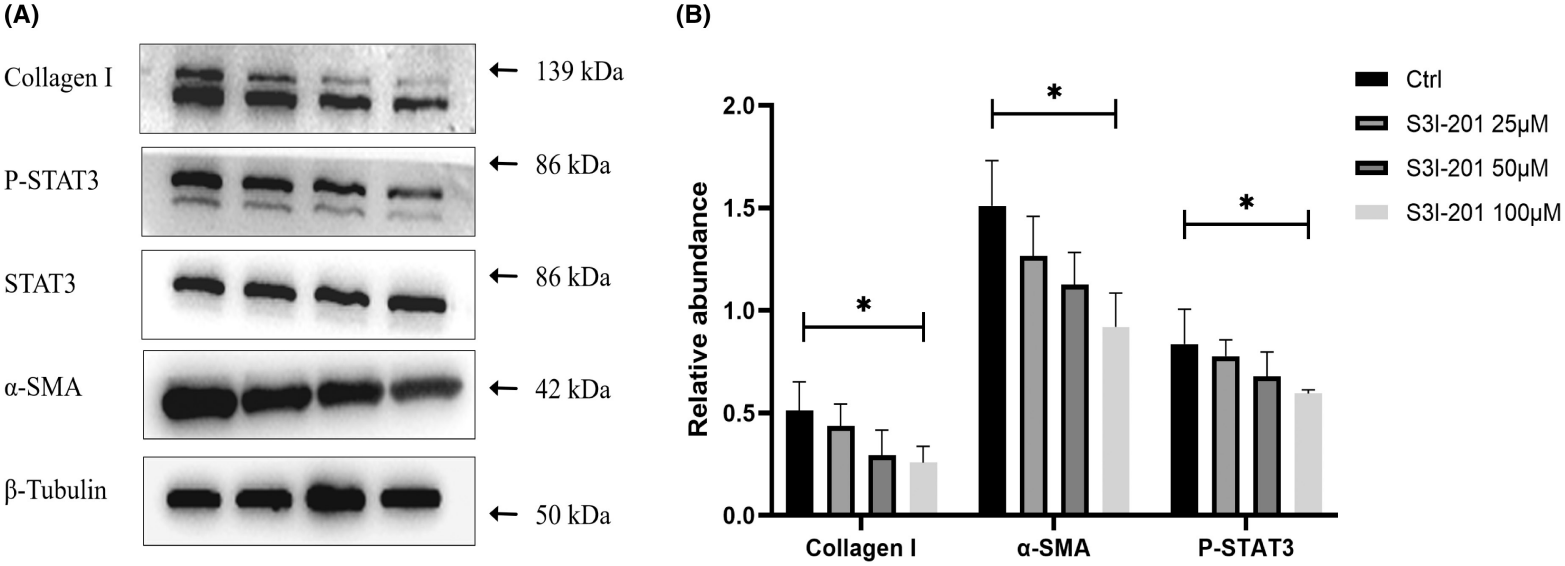
Effects of different concentrations of S3I‐201 on the expression of collagen‐I and α‐SMA in RPFBs. *p<0.05; **p<0.01.

We further investigate the effect of STAT3 inhibitor S3I‐201, a chemical inhibitor which binds to the STAT3–SH2 domain to block dimerization of STAT3, STAT3 DNA binding and transcription activity on the myofibroblast differentiation and collagen I synthesis in RPFBs. As shown in Figure 2, S3I‐201 treatment induced down‐regulation of collagen‐I, p‐STAT3 and α‐SMA in a dose‐dependent manner.

### 3.3 Inhibition of STAT3 suppressed fibroblast proliferation and myofibroblast differentiation induced by TGF‐β1

**FIGURE 3:**
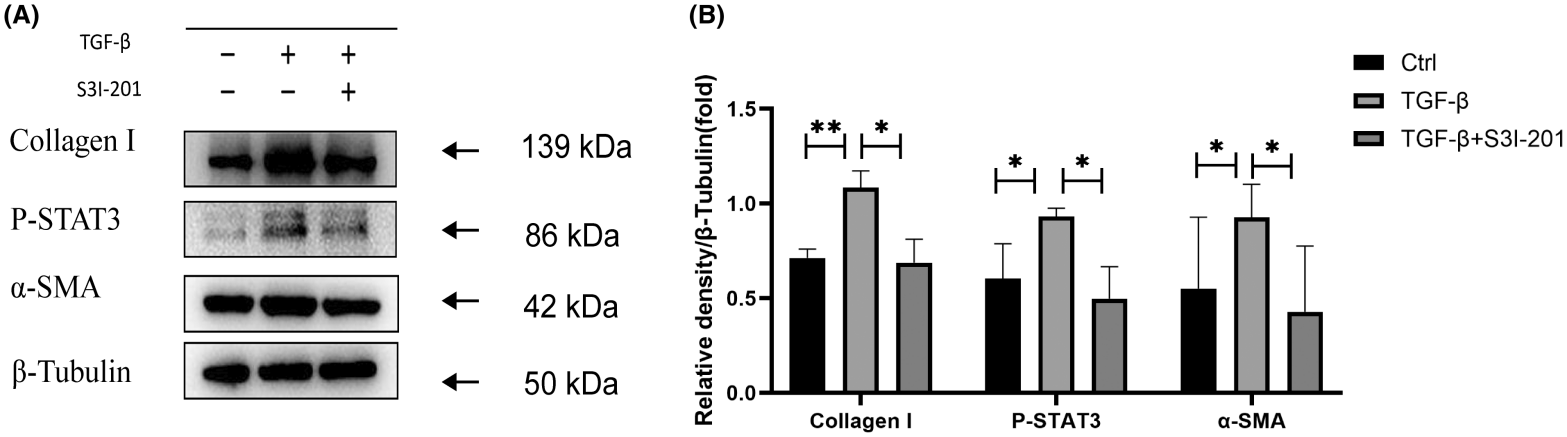
Increased expression of collagen‐I, α‐SMA and P‐STAT3 induced by TGF‐β1 was suppressed by S3I‐201, as assessed by western blot. *p<0.05; **p<0.01.

The activation of fibroblasts to transform into α‐SMA positive myofibroblasts has been considered as a key step of progressive fibrosis. To investigate the relationship between STAT3 activation and phenotype conversion in RPFBs, the RPFBs were first treated with transforming growth factor (TGF)‐β1 for 24 h, the protein level of collagen‐I, α‐SMA and P‐STAT3 were determined. As shown in Figure 3A,B, TGF‐β1 increases synthesis of α‐SMA and collagen‐I in RPFBs, with increased expression of phosphorylation of STAT3. S3I‐201 treatment was able to reduce the synthesis of α‐SMA and the collagen‐I, and markedly decreased phosphorylation of STAT3 in fibroblasts induced by TGF‐β1.

### 3.4 STAT3 induced by IL‐6 was associated with myofibroblast differentiation and increased collagen‐I synthesis of RPFBs

**FIGURE 4:**
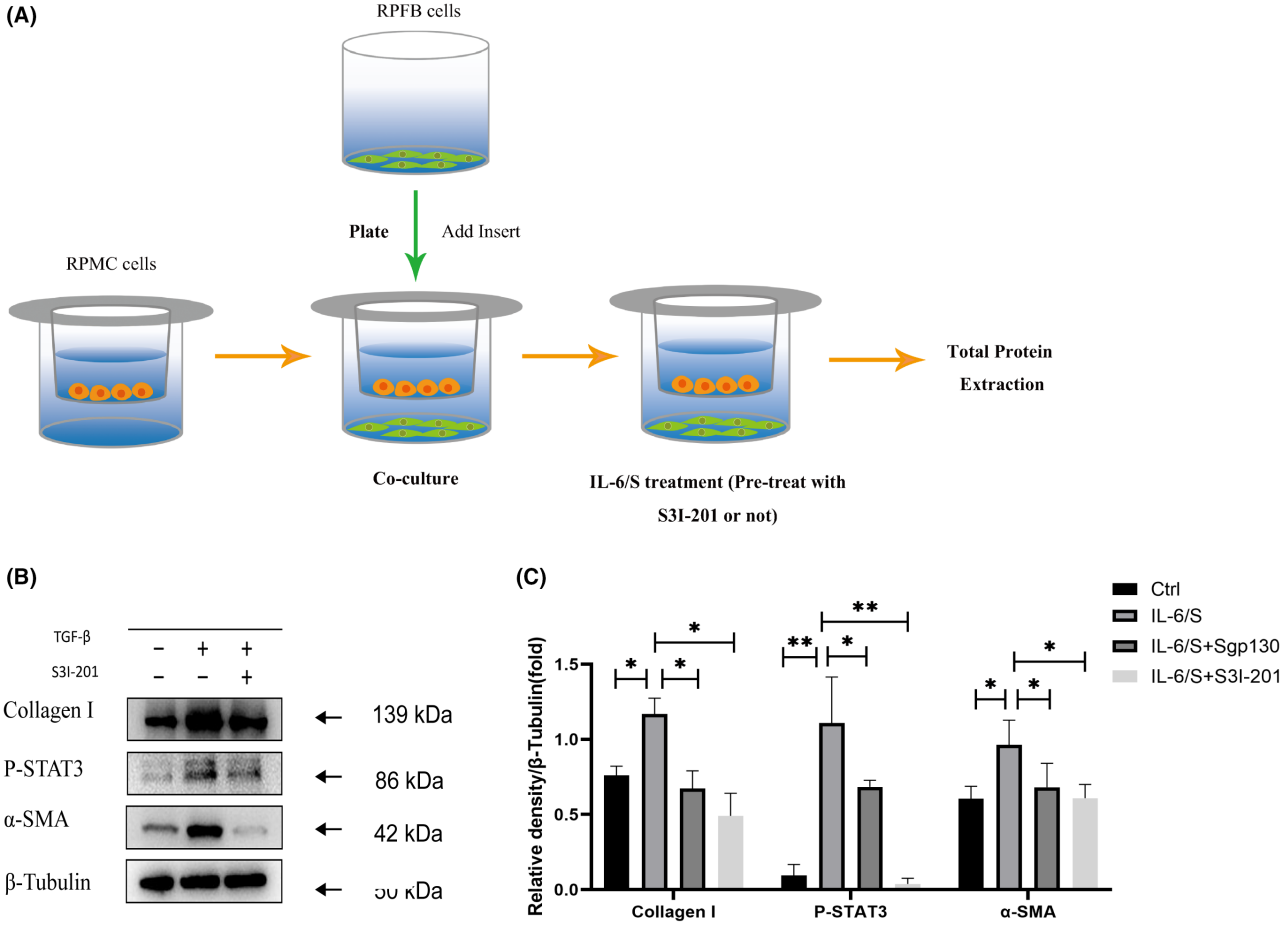
Effects of different concentrations of S3I‐201 on the expression of collagen‐I and α‐SMA in RPFBs. *p<0.05; **p<0.01.

To mimic the in vivo environment, we established a co‐culture system between RPMCs and RPFBs showing as Figure 4A. We next examined the effect of STAT3 activation on collagen‐I synthesis and myofibroblast differentiation on RFFBs in the co‐culture system induced by IL‐6. RFFBs were divided into four groups as follows: Negative control (Ctrl) group; IL‐6/S group; IL‐6/S + sgp130 group; IL‐6/S + S3I‐201 group. The IL‐6/S group was incubated with the IL‐6/sIL‐6R complex for 24 h. The IL‐6/S + sgp130 group was incubated with IL‐6/sIL‐6R complex and sgp130 for 24 h. The IL‐6/S + S3I‐201 group was incubated with IL‐6/sIL‐6R complex and S3I‐201 for 24 h. Then expression levels of Collagen‐I, p‐STAT3, and α‐SMA protein in RPFBs were detected. As seen in Figure 4B,C, in the co‐culture system, IL‐6/S significantly increased expression of collagen‐I, α‐SMA and p‐STAT3 in RPFBs. Treatment with sgp130 or S3I‐201 significantly blocked STAT3 phosphorylation and suppressed increased expression of collagen‐I and α‐SMA induced by IL‐6/S. These data suggested that after incubated with IL‐6/S, STAT3 activation in RPFBs was associated with increased expression of collagen‐I and myofibroblast differentiation in fibroblasts.

### 3.5 Pharmacological inhibition of STAT3 prevented CG‐induced peritoneal fibrosis in mice

**FIGURE 5:**
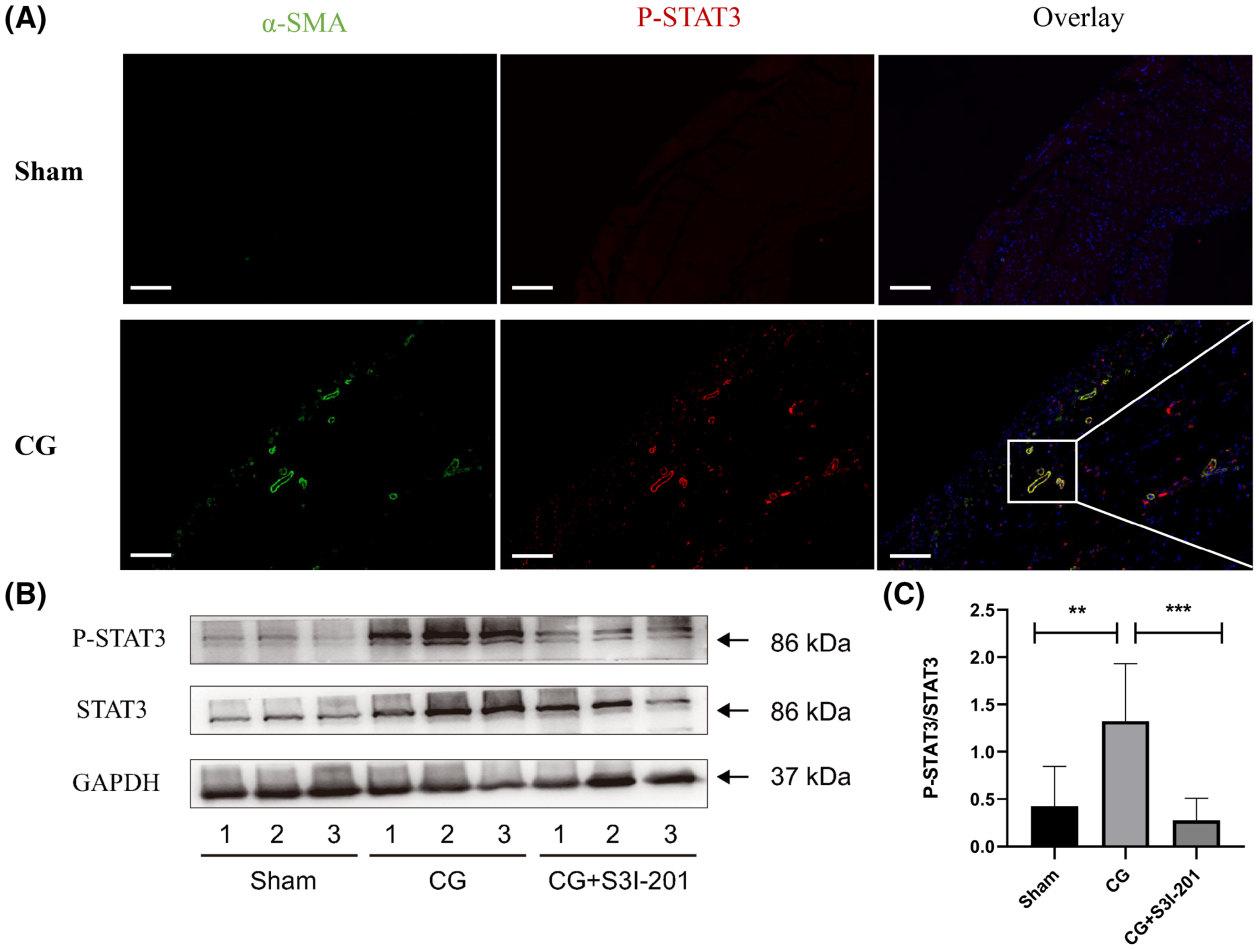
The activation of STAT3 signalling in mouse peritoneal fibrosis model. (A) P‐STAT3 (green) co localized with α‐SMA (red) in submesothelial area in CG‐induced peritoneal fibrosis. (B, C) Expression levels of p‐STAT3, STAT3, or GAPDH in peritoneum lysates were subjected to immunoblot. Results were representative three samples. and the values were expressed as the mean ± SE of three experiments. *p*‐values resulted from t tests. **p<0.01; ***p<0.001.

To explore whether STAT3 activation contributed to peritoneal fibrosis, a mouse peritoneal fibrosis model was established by exposing the peritoneal membrane to CG that induces aseptic peritoneal inflammation with peritoneal membrane damage, leading to tissue fibrosis.^22^, ^23^ Immunofluorescence showed that in the fibrotic peritoneum of CG‐treated mice, α‐SMA‐positive fibroblasts were mainly observed in sub‐mesothelial area of the fibrotic peritoneum and P‐STAT3 positive fibroblasts were found localized among α‐SMA‐positive cells (Figure 5A), suggesting a role for STAT3 signalling in fibroblasts activation. Immunoblot results showed the increased expression of P‐STAT3 significantly in CG‐challenged mice as compared to control mice. However, the increase in peritoneal expression of P‐STAT3 induced by CG was suppressed by S3I‐201 treatment (Figure 5C). Our results suggested that STAT3 signalling played an important role in myofibroblast differentiation of peritoneal fibrosis.

**FIGURE 6:**
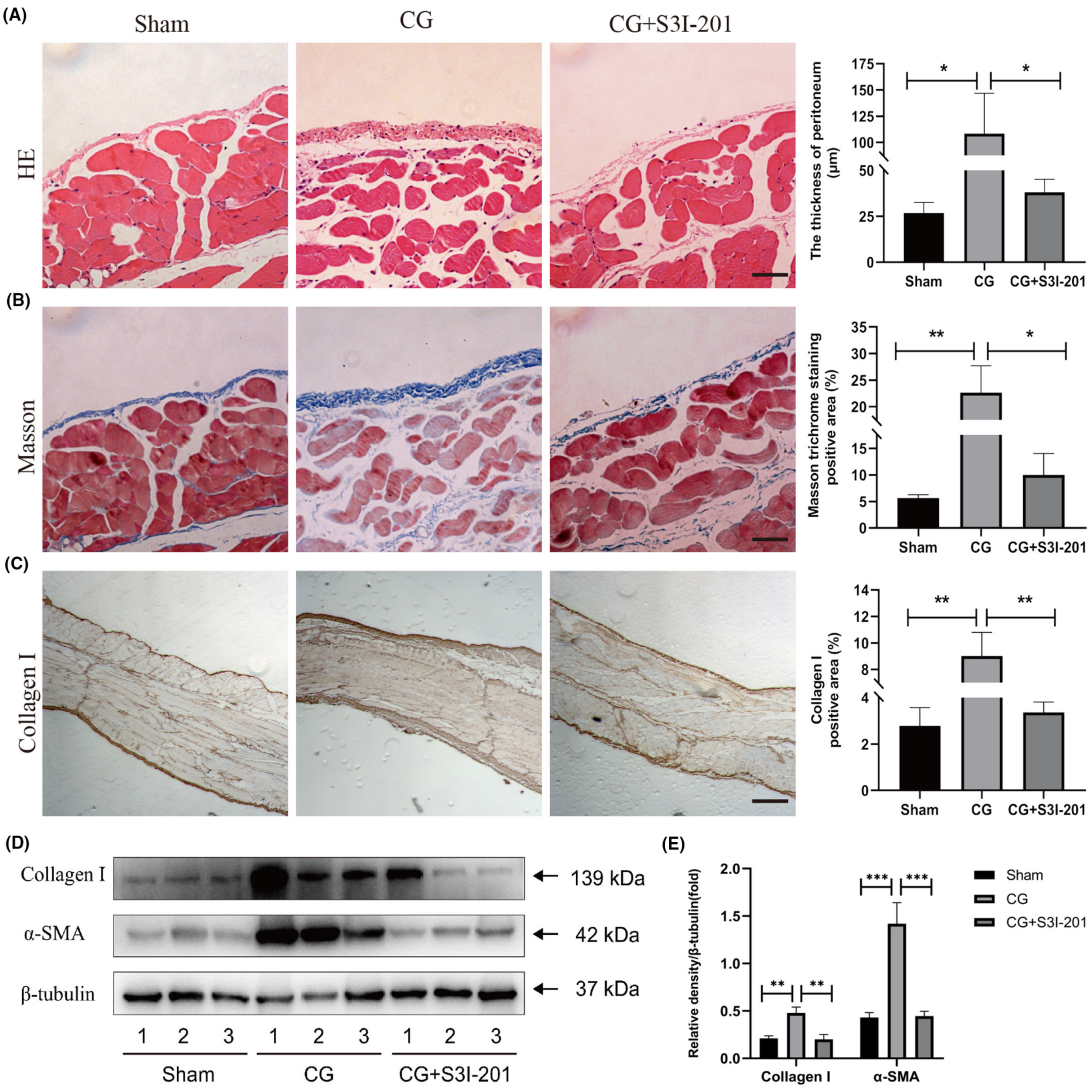
S3I‐201 administration significantly induced reduction of the peritoneal thickness, and collagen‐I‐positive area in mice treated with CG. (A) haematoxylin and eosin staining, (B) Masson and (C) IHC staining of mice parietal peritoneum. (D‐E) S3I‐201 markedly decreased synthesis of Collagen I and a‐SMA in fibroblasts induced by CG, as assessed by western blot. *p<0.05; **p<0.01; ***p<0.001.

We used S3I‐201 to investigate whether inactivation of STAT3 played an anti‐fibrotic role in mouse peritoneal fibrosis model. The thickness of submesothelial compact areas significantly increased while collagen deposition was greater in the CG‐treated mice when compared with those receiving sham treatment, evidenced by haematoxylin and eosin staining, Masson trichrome staining or immunostaining. As shown in Figure 6A–C, S3I‐201 administration to CG‐injected mice significantly decreased the peritoneal thickness and deposition of collagen fibrils. In parallel to these morphological protective effects, the Western blot results showed that CG injections markedly increased the expression of collagen‐I and α‐SMA when compared to that in the control mice, and the increase in peritoneal expression of collagen‐I and α‐SMA observed in CG‐injected mice was significantly blunted in CG‐injected S3I‐201‐treated mice. These findings indicated that inhibition of STAT3 activation could suppress CG‐induced peritoneal fibrosis.

## 4 DISCUSSION

The major finding of this study was that effect of STAT3 in fibroblast and inhibition of STAT3 exerted protective functions against the peritoneal fibrosis. First, we found inhibition of STAT3 in RPFBs suppressed collagen‐I synthesis and α‐SMA expression and S3I‐201 could inhibit TGFβ‐induced myofibroblast differentiation and ECM overproduction. Next, we established that STAT3 induced by IL‐6/S in a co‐culture system between RPMCs and RPFBs. In the co‐culture system with the MCs and fibroblasts, IL‐6/S treatment induced myofibroblastic differentiation and collagen release in fibroblasts, while blocking STAT3 with sgp130 or S3I‐201 strongly ameliorates IL‐6/S‐induced fibrosis. Finally, treatment with S3I‐201 effectively attenuated increased thickness of peritonea membrane in parallel with reduced α‐SMA expression and collagen‐I synthesis that occurred in the peritoneal fibrosis group. Therefore, blockade of STAT3 signalling might be a potential treatment strategy in peritoneal fibrosis.

The role of transforming growth factor‐β (TGF‐β) in the development of fibrosis has been well clarified. Indeed, induction of TGF‐β synthesis in the peritoneum by adenovirus‐mediated gene transfer has been reported to induce progressive thickening of peritoneal membrane, increased myofibroblastic differentiation, and abnormal production of ECM,^24^, ^25^ whereas direct blockade of TGF‐β by synthetic peptides prevents peritonea membrane injury.^26^ It has been demonstrated that TGF‐β could activate STAT3, and TGFβ‐induced fibroblast‐to‐myofibroblast transition and collagen release were inhibited by pharmacological or genetic inactivation of STAT3 in cultured fibroblasts.^27^ Xu et al. found that STAT3 over‐activation can up‐regulate the expression of TGF‐β1 in liver tissues of chronic hepatitis B patients and liver fibrosis model of rats and cooperate with TGFβ1 to aggravate the damage and fibrosis of liver tissue.^28^ Pang et al. showed that rat renal interstitial fibroblasts lacking STAT3 are not sensitive to TGF‐β‐induced fibrosis, and S3I‐201 can be time‐ and dose‐dependent inhibit the expression of fibronectin (FN) and α‐SMA in rat renal interstitial fibroblasts.^11^ In the study by Chakraborty et al., the authors reported that STAT3 induced dermal fibroblasts to differentiate into myofibroblasts by integrating common profibrotic pathways, including upstream kinases SRC, c‐ABL, JAK2, and JNK.^13^ Study on the burn‐induced hypertrophic scarring also showed that IL‐6 trans‐signalling could induce activation of STAT3 and promoted fibroblast proliferation and ECM synthesis in scar tissue.^29^ Similarly, in the present study, we found that pharmacological inactivation of STAT3 in cultured RPFs prevents TGF‐β‐induced myofibroblast differentiation and significantly decreases the TGF‐β‐stimulated collagen release, suggesting the important role for STAT3 in driving peritoneal fibrosis.

We also found that in the co‐culture system, stimulation of fibroblasts with IL‐6/S induces an activated myofibroblast phenotype and collagen release, and inhibition of STAT3 with sgp130 or S3I‐201 suppressed the transdifferentiation of fibroblasts with impaired upregulation of α‐SMA protein and reduced release of collagen‐I protein. These results indicated that MCs and fibroblasts may contributed to peritoneal fibrosis via STAT3 activation. IL‐6 trans‐signalling plays an important role in peritoneal fibrosis. In our previous work, we demonstrated that that IL‐6 trans‐signalling activates STAT3 in HPMCs and participates in the MMT process to promote peritoneal fibrosis.^8^

Myofibroblasts are major source of ECM in physiological tissue repair and have been demonstrated to responsible for increased production of ECM in tissue fibrosis. Overwhelming evidence highlights that in fibrotic diseases, resident fibroblasts are activated and develop a persistently activated α‐SMA‐positive myofibroblastic phenotype and produce abnormal ECM in fibrotic tissues.^4^, ^5^, ^29^ Therefore, targeting signalling pathways that mediate fibroblasts activation may be one approach to attenuate fibrosis progression. STAT3 has been well known as a regulator of fibroblasts survival, proliferation, activation and a potential molecular therapeutic target in fibrosis. S3I‐201 is a selective inhibitor of STAT3 activity, which binds to the SH2 domain of STAT3 to blocks dimerization of STAT3, STAT3‐DNA binding and transcriptional activities.^14^ S3I‐201 preferentially interacts with STAT3 monomer and has a low toxicity to cells with no aberrant STAT3.^14^ These properties are essential for the treatment of peritoneal fibrosis. S3I‐201 has been demonstrated to ameliorate kidney and lung fibrosis.^11^, ^16^ It has been shown that STAT3 can significantly reduce renal damage in HIV‐related nephropathy, diabetic nephropathy or obstructive nephropathy, while knockout STAT3 gene can significantly reduce fibroblast proliferation and ECM synthesis, ameliorates renal interstitial fibrosis.^30^ Chakraborty et al. reported that mice lacking STAT3 selectively in fibroblasts are less sensitive to TBRIact‐induced fibrosis.^13^ Previous studies have suggested that blocking JAK2, an upstream activator of STAT3, by ruxolitinib can reduce peritoneal mesothelial cell injury, inflammation, fibrosis and angiogenesis induced by non‐biocompatible peritoneal dialysate.^31^ Recent studies have found that gefitinib, an inhibitor of EGF receptor (EGFR), can significantly improve peritoneal fibrosis in experimental mouse models by inhibiting STAT3 phosphorylation.^21^ Consistent with these results, in our study, we found that S3I‐201 administration significantly attenuated peritoneal fibrosis in mice, as evidenced by down‐regulation of Collagen I and α‐SMA expression with inhibited phosphorylation of STAT3. Furthermore, colocalization of P‐STAT3 and α‐SMA was confirmed by immunofluorescence double staining in the peritoneal fibrosis group. These observations correspond to the research results of Kuratsune et al.,^32^ and Pang et al.^11^ indicating that p‐STAT3 preferentially colocalizes with α‐SMA in the tubulointerstitium of the rat kidney. Given the co‐localization among α‐SMA and P‐STAT3 and decrease in fibrosis with inhibition of STAT3 in the mouse peritoneal fibrosis model, our findings suggest that STAT3 play a vital role in fibroblasts activation during peritoneal fibrosis.

However, the conclusions from our study are challenged by several limitations. First, we used primary rat peritoneal mesothelial cells (RPMCs) and fibroblasts (RPFBs) rather than primary human peritoneal mesothelial cells and fibroblast. Also, we used intraperitoneal injection of CG rather than peritoneal catheter instillation of high glucose‐based dialysate to induce peritoneal fibrosis in mice. Finally, we lacked evidence of the relationship between peritoneal fibrosis and STAT3 phosphorylation in PD patients, which will be supplemented and improved in our further studies.

## 5 CONCLUSIONS

Taken together, these findings suggested that STAT3 contributed to TGFβ‐induced and IL‐6/S‐induced activation of fibroblasts and was involved in progressive peritoneal fibrosis. Pharmacological inhibition of STAT3 markedly attenuate peritoneal fibrosis and STAT3 can be a potentially effective antifibrotic molecular‐target.

## AUTHOR CONTRIBUTIONS

**Qianhui Song:** Conceptualization (equal); investigation (equal); validation (equal); writing – original draft (equal). **Han Li:** Investigation (equal); validation (equal). **Hao Yan:** Formal analysis (equal). **Zanzhe Yu:** Formal analysis (equal). **Zhenyuan Li:** Formal analysis (equal). **Jiangzi Yuan:** Formal analysis (equal). **Na Jiang:** Formal analysis (equal). **Zhaohui Ni:** Writing – review and editing (equal). **Leyi Gu:** Writing – review and editing (equal). **Wei Fang:** Conceptualization (equal); writing – review and editing (equal).

## FUNDING INFORMATION

This work was supported by the National Basic Research Program of China (grant no. 81370864, 81670691) and Shanghai Municipal Education Commission‐Gaofeng Clinical Medicine (grant no. 20152211).

## CONFLICT OF INTEREST STATEMENT

No conflicts of interest, financial or otherwise, are declared by the authors.

## Supporting information


Figure S1‐S2.

## Data Availability

The authors confirm that the data supporting the findings of this study are available in the methods and supplementary material of this article.
