# Correction to “Inhibition of STAT3 by S3I‐201 Suppress Peritoneal Fibroblast Phenotype Conversion and Alleviate Peritoneal Fibrosis”

**DOI:** 10.1111/jcmm.71115

**Published:** 2026-04-21

Song Q, Li H, Yan H, et al. Inhibition of STAT3 by S3I‐201 Suppress Peritoneal Fibroblast Phenotype Conversion and Alleviate Peritoneal Fibrosis. *Journal of Cellular and Molecular Medicine*. 2024;28:e18381. doi:10.1111/jcmm.18381

In the article, there were errors in figure citation, Figure 4 and legend.

In page 4, Results, first paragraph, the citation of Figure 3B,D is incorrect. This should be Figure 1B,D.

Figure 4B and Figure 4 legend are incorrect. The correct image and legend are shown below:

**FIGURE 4:**
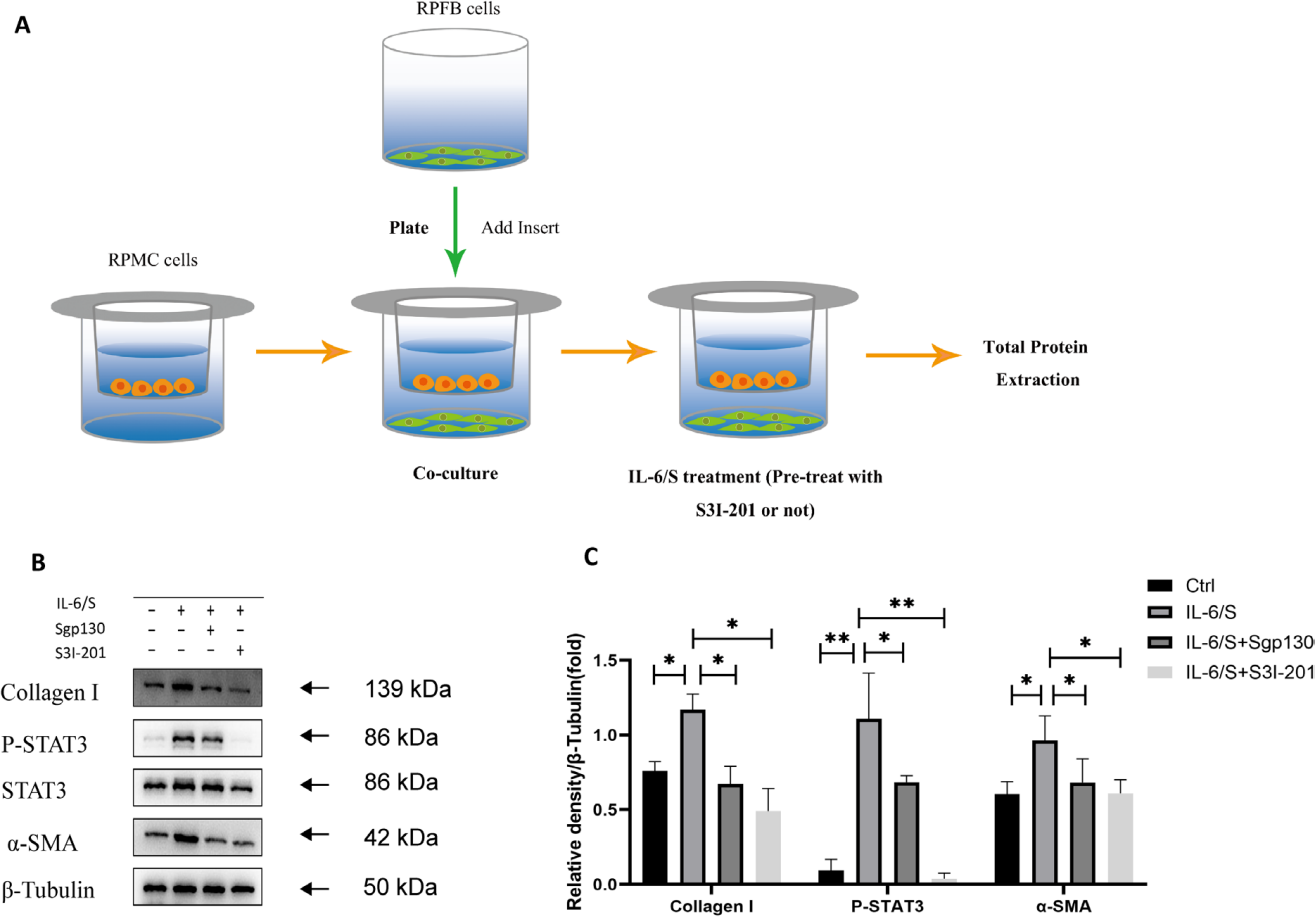
S3 I‐201 inhibits the myofibroblast differentiation and collagen synthesis of RPFB induced by IL‐6/S in the co‐culture system.

