# Supplementary material for: Inhibition of STAT3 by S3I‐201 suppress peritoneal fibroblast phenotype conversion and alleviate peritoneal fibrosis
Source: J Cell Mol Med. 2024 May 23;28(10):e18381. doi: 10.1111/jcmm.18381 (PMC11114217; doi:10.1111/jcmm.18381)
Supplement: Supplementary file 1 — Figure S1‐S2. [file JCMM-28-e18381-s001.docx]

# supplementary material


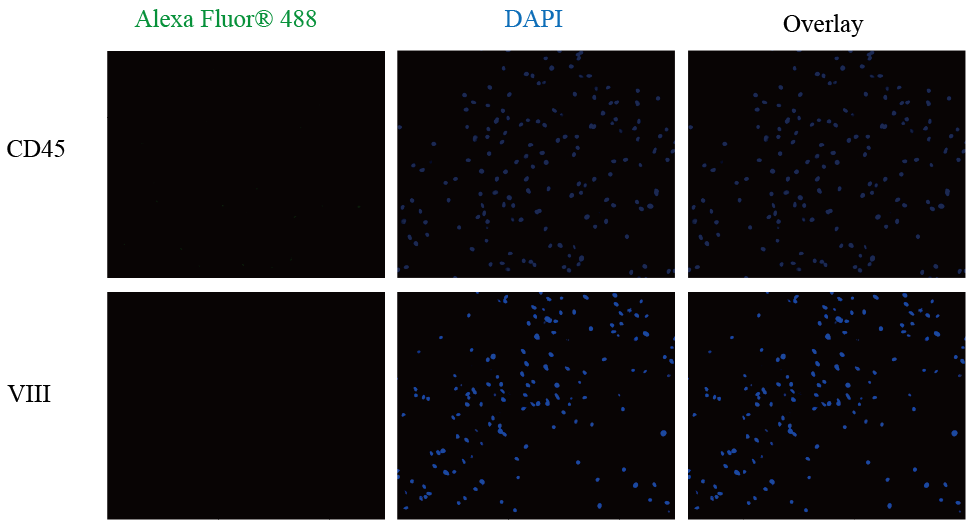


**Figure S1.** Identification of primary cultured rat peritoneal mesothelial cells (200×). The peritoneal mesothelial cells showed negatively expressed CD45 and factor VIII.


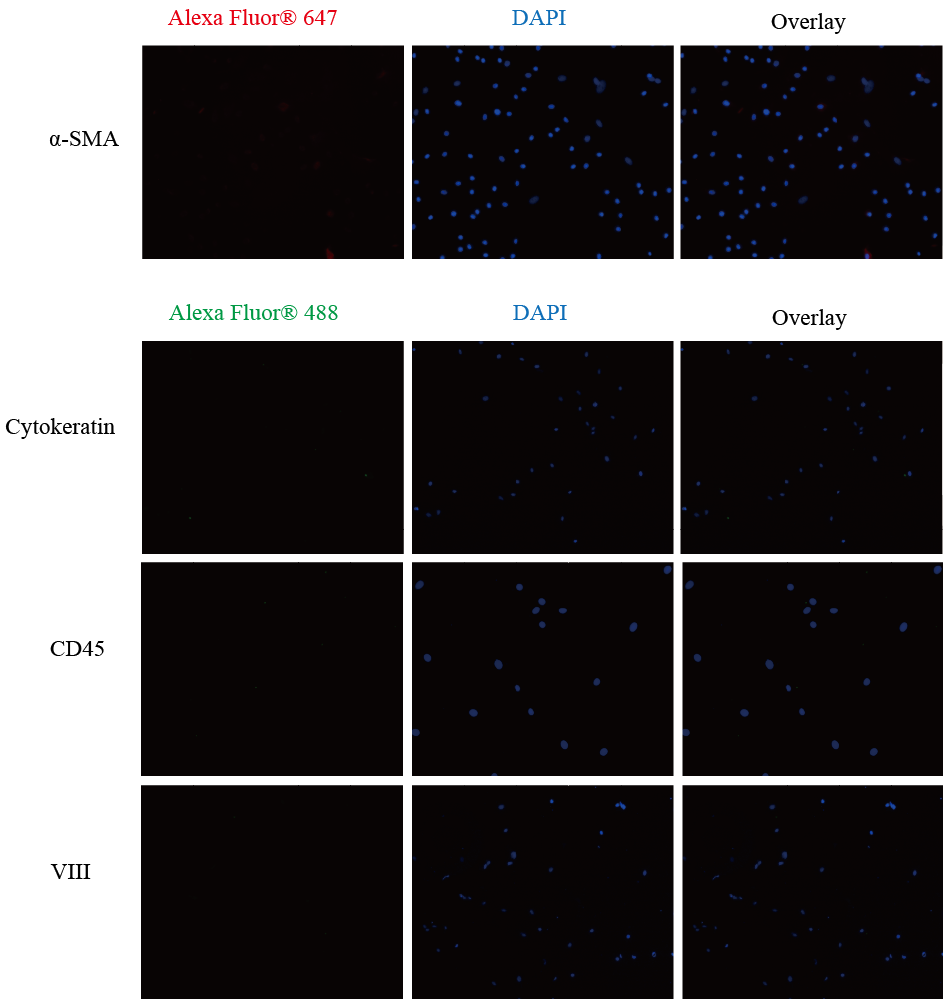


**Figure S2.** Identification of primary cultured rat peritoneal fibroblasts (200×). The peritoneal fibroblasts were negative for α-SMA, CD45, cytokeratin and factor VIII.
